# Intracranial hypertension and deep sedation

**DOI:** 10.1186/s13054-019-2578-3

**Published:** 2019-11-04

**Authors:** Guillermo Bugedo, César Santis

**Affiliations:** 1 Departamento de Medicina Intensiva, Pontificia Universidad Catolica de Chile, Marcoleta 367, 6510260 Santiago, Chile; 2 Unidad de Pacientes Críticos, Hospital Barros Luco Trudeau, Santiago, Chile

To the editor

We read with interest the article by Robba and Citerio on the management of intracranial hypertension, and we agree with the need to take images to rule out surgical options, secondary to space-occupying masses or hydrocephalus [1]. However, we differ from some first-line options suggested by the authors, such as intensifying sedation with propofol in doses of 4 to 6 mg/kg/h.

First, the impact of deep sedation in neurocritical patients is debatable as the evidence is weak [2–4]. The main problem during intracranial hypertension is not an increase in oxygen consumption, which could be solved with deeper sedation, but the decrease in cerebral blood flow and tissue perfusion. Although propofol can lower intracranial pressure, it is a potent cardiovascular depressant and it may require high-dose vasopressors to maintain perfusion pressure at acceptable levels [5]. In particular, propofol infusion syndrome is a catastrophic situation that is described in doses greater than 4 mg/kg/h [2].

Beyond the multiple reports that show that deep sedation in the general population of critically ill patients on mechanical ventilation is associated with worse outcomes, in the neurocritical patient deep sedation eliminates the possibility of assessing the clinical response [2]. Thus, we prefer to use propofol in doses not exceeding 3 mg/kg/h, which reduces oxygen consumption and have an anticonvulsant effect [2], along fentanyl 1-2 μg/kg/h to facilitate adequate synchrony with the ventilator. In this setting, propofol infusion can be temporarily suspended and clinical response evaluated, which may help in the decision making process.

Second, facing an intracranial hypertension crisis and before increasing sedation, it is essential to assess preload using dynamic predictors, or a simple measurement of central venous pressure. Many of these patients receive or have received hypertonic fluids and may be in a state of hypervolemia. Facing intracranial hypertension, a state of preload dependence while maintaining macro hemodynamic parameters may be desirable to favor venous drainage from the cranial vault.

Third, the analysis of ventilation and airway pressures must be carefully analyzed before intensifying sedation. Again, venous return from the cranial vault can be facilitated by lowering the PEEP level if there is obvious overdistention, or working the ventilator for synchrony.

Finally, deep sedation should be seen as a third-line measure in cases of refractory intracranial hypertension, and as an alternative or a bridge to decompressive craniectomy, as Robba and Citerio clearly commented.

## Authors' response

In a setting in which many of our therapeutic strategies are based on weak evidence [3], the variation of care is the rule [6, 7] and we described our management strategy [1].

We increase propofol if EEG derived parameters do not indicate a deep suppression of metabolism. The metabolism reduction induces a fall of cerebral blood flow (metabolic coupling) and therefore an ICP reduction. In many patients, this optimization of sedation is sufficient for controlling ICP. On the other hand, if metabolism is already suppressed, we do not increase propofol.

We are aware that this strategy has circulatory and metabolic side effects. Usually, the augmentation of propofol dose is associated with an increment of vasopressors aimed to maintain optimal CPP.

The propofol infusion syndrome development is monitor and if early signs present, we stop propofol.

Euvolemia, fluid balances, respiratory parameters and PEEP are continuously monitored, and their optimization is part of the general neuroICU practice.

Recently this strategy has been inserted in the “Algorithm for Patients with Intracranial Pressure Monitoring: The Seattle International Severe Traumatic Brain Injury Consensus Conference" [8], result of the extensive work of a panel of 10 neurointensivists, 23 neurosurgeons, 5 neurologist/neurointensivists, 2 trauma surgeons, 2 Emergency medicine specialists. Therefore, this strategy is not only ours but recognized by a large group of experts.

## Data Availability

Not applicable.
